# International regional competitiveness of rural territories as a factor of their socio-economic development: Methodological aspects

**DOI:** 10.1016/j.heliyon.2023.e23795

**Published:** 2023-12-17

**Authors:** Vitaliy Kovshov, Milyausha Lukyanova, Zariya Zalilova, Oksana Frolova, Zagir Galin

**Affiliations:** Department of Economics and Management, Federal State Budgetary Educational Establishment of Higher Education “Bashkir State Agrarian University”, Ufa, Russia

**Keywords:** Agro-industrial complex, Agribusiness, Competitive advantages, Competitiveness, Food export, Foresight, Production potential

## Abstract

The study aims to develop a scientific and methodological approach to the formation of a model for managing the international competitiveness of rural areas based on an assessment of the existing export and production potential to form priorities for their socio-economic development. Research methods involve a systematic approach, an integrated approach, an algorithmization using methods of integral assessment of competitiveness, etc. The research allowed the development of a conceptual model for managing the international competitiveness of rural regions. The model includes a variable list of determinants and indicators of the formation of competitive advantages of rural territories depending on the export-production potential and strategic goals of their development. The paper presents a model management algorithm and a set of complementary methodological techniques used to assess international and regional competitiveness. The scientific novelty lies in the developed scientific and methodological approaches and practical recommendations for the formation of international and regional competitiveness in rural areas. Moreover, a system of determinants of the competitiveness formation of rural territories and a system of indicators for its assessment have been developed.

## 1 Introduction

Modern trends in the development of the global and regional economy with pronounced external challenges in the background provide a rationale for the strategic planning of new “growth points” in rural areas competitive in sub-regional, national and foreign markets. The issue of socio-economic development planning of rural areas is urgent in countries with low fiscal capacity of rural areas and a high level of differentiation of the production and social potential of rural regions. Agriculture, product processing and agribusiness in general are undoubtedly the main drivers and “points of development” of rural areas. However, the choice of drivers and strategies for the rural development of certain regions in Europe and Asia should be based on a competitiveness assessment of the region in individual national and global agri-food markets considering their production, export potential and determinants of the sustainable competitiveness development [[1], [2], [3]].

One of such countries is the Russian Federation. The number of rural population in the country is 25.3 %. The differentiation by region is very high - from 70.7 % in the Altai Republic to 3.9 % in the Magadan region. The share of agriculture, forestry, hunting, fishing and fish farming in the gross value added of Russia is 4.1 % (from 27.3 % in the Kamchatka Territory to 0.6 % in the Tyumen region). The degree of differentiation by soil and climatic conditions in the country is high (from Arctic to temperate continental). Besides, the share of the rural population has decreased by 0.6% points over the past 5 years. The share of agriculture in the gross value added of the country has also decreased [4].

Strategic planning of rural development based on the assessment of the national and international competitiveness of the region makes it possible to identify top export-oriented directions of the agro-industrial complex development for specific rural areas acting as “poles of socio-economic growth”. The assessment of the rural areas' export and production potential, the level of competitiveness of the region and the influence of key competitiveness determinants allows identifying the most favorable rural areas as “potential growth points” of the agricultural sector and the drivers of rural development of a particular region.

In Germany, where the active promotion of competitive rural areas has resulted in increased employment, improved living standards, and the sustainable development of regions, this example underscores how international experience can be applied to rural development [5].

Furthermore, research demonstrates that Finland has successfully formulated and implemented strategies focused on rural region development through rural entrepreneurship and innovation. For instance, local entrepreneurship support programs and financial incentives for innovation have contributed to increased employment and job creation in rural areas. Canada has adopted targeted initiatives aimed at fostering the agricultural sector and the growth of rural regions. Farmer support programs, agricultural product export promotion, and infrastructure investments have aided in enhancing the competitiveness of Canadian rural territories. Switzerland is renowned for its effective rural region development policy, which is grounded in support for rural tourism, natural resource preservation, and the promotion of local production. This has not only bolstered the economies of rural regions but also preserved their unique characteristics [[6], [7], [8]].

Therefore, the assessment and management of competitiveness, the realization of the export agro-food potential of individual countries and rural areas, is becoming a global and relevant topic of scientific and practical research scientists around the world.

The development of the competitive institutional environment, global competitive relations, and the intensification of competitive behavior of individual regions in all parts of the world create a need for fundamental study of the determinants of the formation of regional competitiveness, the improvement of adapted methodological tools for the formation of international, regional competitiveness of rural areas and the development of practical recommendations to ensure sustainable competitive advantages.

The problem is reinforced because opinions on the methodological apparatus for assessing international and regional competitiveness and the priority of sources of its formation differ. Algorithmization of mechanisms for managing the competitiveness of rural regions is insufficient. Besides, state support for the export of products by local producers has a polemical character. In the current conditions, a systematic approach to the methodological tools of the international competitiveness of regions based on the science genesis study, economic thought and practice becomes a significant motivator for the research. The formation of a sustainable competitive advantage of rural areas in foreign markets should be based on a clear algorithmization of the process, considering the determinants of the competitive environment, building trends in the competitiveness indicators, and using instrumentally adapted models for managing international and regional competitiveness.

The study aims to develop a scientific and methodological approach to the formation of a model for managing the international competitiveness of rural areas based on an assessment of the existing export and production potential to form priorities for their socio-economic development.

In accordance with the study purpose, the key areas of research are as follows.

- to develop a model for managing the international and regional competitiveness of rural areas based on integrated and systematic approaches considering the production and export potential;
- to form subsystems of determinants and indicators of the competitive advantages formation of rural areas in the model to make it possible to adapt in different countries of the world depending on institutional conditions and strategic goals of socio-economic development;
- to develop methodological approaches to the formation of high international competitiveness in rural areas of the world, to carry out instrumental adaptation on the example of a number of regions, and to propose a set of measures to ensure their sustainable competitive advantage.

Thus, research in the field of international rural competitiveness stands out for its innovative approach, grounded in the concept of ‘territorial clusters of sustainable development.’ This conceptual framework enables the integration of the territorial characteristics and resources of rural regions with the principles of sustainable development and competitiveness.

We propose territorial clusters as a tool for the balanced development of rural areas, where production and environmental aspects interact, taking local characteristics into account. This approach takes into consideration the region's potential and enables the creation of innovative models for the production and export of agricultural products.

The theoretical foundation of our position is rooted in the literature on sustainable development, the concept of territorial clusters, and competitiveness theory. We aim to illustrate how this integrated approach can effectively govern the international competitiveness of rural areas, thereby ensuring sustainable socio-economic development.

## 2 Literature review

Scientific, methodological and practical aspects of the formation and assessment of the international competitiveness of regions, and the choice of strategies for managing their competitiveness in various conditions are the subject of research by many well-known scientists. At the same time, there are various methodological and organizational approaches to the study of this problem.

Researchers note that international competitiveness is commonly analyzed at three levels - countries, firms and individuals [1]. Assessment of international competitiveness in agricultural trade is mainly studied at the country level. At the same time, research focuses on volume, sales structure, or comparative advantages [2]. The same methodological approaches are often used to assess either the region or country's competitiveness. The main results show five well-studied, three new and two insufficiently studied theories, while quantitative research is the main method of analysis [3].

Indicators play a significant role in assessing competitiveness. The paper's authors consider the problems of choosing criteria for assessing international competitiveness (indicators) to be the most controversial. The International Market Share Index (IMS), the Trade Competitiveness Index (TC) and the index of revealed comparative advantages (RCA) are the most often used evaluation indicators [4,5]. As criteria for food production export competitiveness, Polish scientists used the indicators of the share in the world market, the coefficient of comparative advantage, the coefficient of import coverage, the Grubel–Lloyd export index, and the synthetic indicator of international competitiveness. These indicators were the highest in such countries of the European Union as the Netherlands, France, Spain and Denmark [6,7].

Some researchers measure the competitiveness of countries using such trade indicators as the net export index and Wallrath indices. According to these indicators, France and Spain occupied a highly competitive position. The Netherlands and Italy show the best trends in the growth of international competitiveness in agriculture and the food industry [8].

Italian scientists propose a methodology based on two approaches, which consider indicators of similarity and complexity of exported goods, categorized into 95 positions. The analysis shows that there are different groups of countries with different competitiveness. The first group comprises the Czech Republic and Poland, which are in the qualitative catch-up phase and increase their competitiveness. The second group includes Bulgaria, Romania and other countries that are trapped in the low-quality segment of agriculture with declining competitiveness indicators in the European market [[9], [10], [11]]. Malaysian scientists rely upon cognitive theories of values and planned behavior. They propose using a managed integrated model to assess the export competitiveness of entrepreneurs [12,13].

Spanish scientists use the Shift-share methodology supplemented with elasticity to assess the competitiveness of the country's agri-food sector. Having studied these variables, it was revealed that Latvia and Lithuania are the most interesting EU markets, while Cyprus, Malta, Poland, the Czech Republic and Sweden have very remarkable values. Bulgaria, Slovakia, Hungary, Romania, Germany, Austria and the United Kingdom have certain problems with the level of specialization [14]. Similar studies of Southeast Asian countries show that Myanmar and the Philippines have the highest levels of agri-food competitiveness in the world market, while Laos and Cambodia are the most competitive in regional markets [15].

Certain methodological efforts to consider the existing production potential have been made for assessing the international competitiveness of the agro-food complex of Russia. Russia ranks third in the world in land resources, which is the main when determining the prospects of agricultural production. This huge potential has not been harnessed enough so far.

Russia, despite its vast potential in terms of land resources, does not fully harness this potential in agriculture. This means that the country fails to achieve the desired level of competitiveness in the agri-food sector. Low competitiveness can imply that Russian products may be less appealing to global consumers compared to products from other countries, which can hinder their export and agricultural development in the country [[16], [17], [18]].

The issues of assessing and managing the international competitiveness of certain rural territories in a particular country have not been properly explored from a methodological point of view. In this context, an approach to assessing regional competitiveness using cluster algorithms of multidimensional classification should be highlighted. The developed method includes an extensive set of data on such indicators as infrastructure development, industrial production capacity, investment activity, foreign trade development and intensity, socio-economic development, institutional factors, characteristics of technological lag and parameters of innovation activity [19,20].

The method for assessing the competitiveness of agribusiness and export opportunities of the region proposed by Serbian scientists involves the use of the indices of comparative advantages and intra-industry integration. The method has been tested in the rural region of Vojvodina, which has high competitive advantages in crop production, but low ones in animal husbandry. The revealed comparative advantage is proposed to be used as a factor of competitiveness growth in sectors where crop production is a raw material base [21]. Scientists of Kazakhstan have developed a strategy of optimal managerial influence for controlling competitiveness. The strategy ensures the formation of the region's export agricultural potential [[22], [23], [24]].

The method for the formation of international competitiveness should cause the development of a system for managing the competitiveness of rural areas and the development of a set of measures to improve it. However, many studies focus only on the development of current measures to improve international competitiveness based on the assessments carried out. For example, researchers from the Netherlands recommend increasing returns of the scale, improving supply chain management and exploiting cultural differences through innovation to increase agri-food competitiveness [[25], [26], [27]]. Indian scientists suggest increasing investment in technologies with a higher level of logistics infrastructure [28,29]. Developed international logistics chains and an effective choice of export outlets contribute to the growth of regional competitiveness [30]. Therefore, methodological studies on the assessment and management of rural area competitiveness lead to the development of specific methods and strategies that can be successfully applied in regional contexts where the characteristics of agribusiness and export opportunities may vary significantly.

Some scientists consider increasing the agri-food competitiveness and export potential of individual countries and rural regions by improving the determinants correlating with the result - material and technical condition, financial investments, agricultural production economic efficiency, the farms' development level, and the processing level [[31], [32], [33], [34], [35]]. There are also methods for analyzing decisions according to a set of criteria (MCDA), which allows for determining the priority of alternatives to competitive strategies [36]. Iranian scientists have attempted to form a model of competitiveness development, which consists of causal conditions, intermediate conditions, contextual conditions, and export strategy. The model defines dynamic abilities that lead to competitiveness and offers a way to create such abilities [37,38]. Some researchers propose using a multidimensional model of competitiveness based on three latent pillars - compete*,* connect and change. These three pillars reflect the traditional static and dynamic concepts of competitiveness [39].

Studies have shown that some issues of assessing and shaping the international competitiveness of rural areas have not been studied properly and require additional research. These issues involve instrumental adaptation of existing methods of assessing international competitiveness to specific economic and organizational conditions, production and export specialization in the context of the transition from the national to the regional level, methodological aspects of the integrated and systemic development of sustainable international competitive advantages of the region, the formation of an adaptive system of determinants and indicators of international competitiveness of regions in connection with the goals of socio-economic development of rural areas and creating a model for managing the international competitiveness of rural regions.

The existing literature on rural region competitiveness research provides some theoretical foundations and empirical data, but there is a lack of systematically innovative methods for managing the competitiveness of rural areas, particularly considering their specificity and diversity. This study aims to fill this gap by offering an innovative methodological approach.

Consequently, this research is directed at stakeholders, including the academic community, governmental and municipal authorities, as well as leaders of agro-industrial enterprises. These groups have an interest in optimizing the competitiveness of rural areas, as it can have an impact on economic development and societal well-being. The rationale behind this lies in the development of novel practical tools that facilitate the enhancement of rural region development, support the increase in their competitiveness, and contribute to socio-economic growth. Therefore, this study offers innovative and practically applicable tools for managing the competitiveness of rural regions, which holds significant importance for both the academic community and industrial and governmental entities.

Hypotheses of the study. It is assumed that the formation of international regional competitiveness of rural areas can be represented as a system model that includes three interrelated elements - a subsystem of incoming competitiveness factors (determinants), a multi-level procedural subsystem and a subsystem of outgoing indicators for assessing the competitiveness of the region. It is expected that there is a certain algorithm of actions that allows the digitization of the proposed model for a specific rural area and develops optimal measures to influence the controlled determinants to achieve the desired values of indicators.

## 3 Data and methods

### 3.1 Study design

**Fig. 1:**
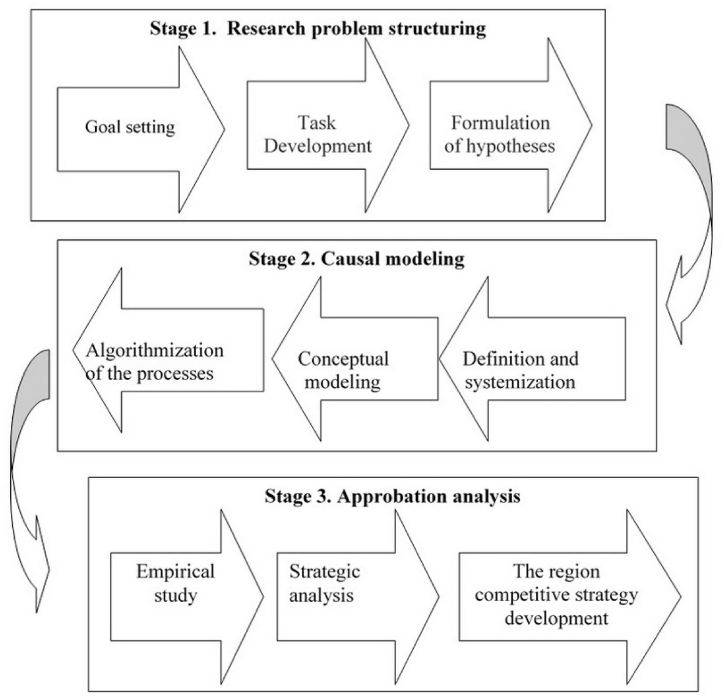
Structure of the research methodology.

*Source:* Authors' development.

The research methodology is based on economic and logical objective laws, socio-economic patterns and fundamental theorems formulated by leading scientists in regional economics and the competitive behavior of territorial entities. A three-stage methodology involving the research problem structuring, causal modelling, and approbation analysis was proposed to achieve this goal (Fig. 1).

The problem structuring stage involves the definition of an actual goal, the development of research objectives and the formulation of hypotheses. This stage of the research implies using some methods. First, the monographic method includes the analysis of the results of previous research by leading world scientists. Second, the goal-setting method involves the development of a general goal and tasks according to the system, strategic settings and the nature of the tasks being solved. Third, the abstract-logical method is used when formulating research hypotheses.

**Fig. 2:**
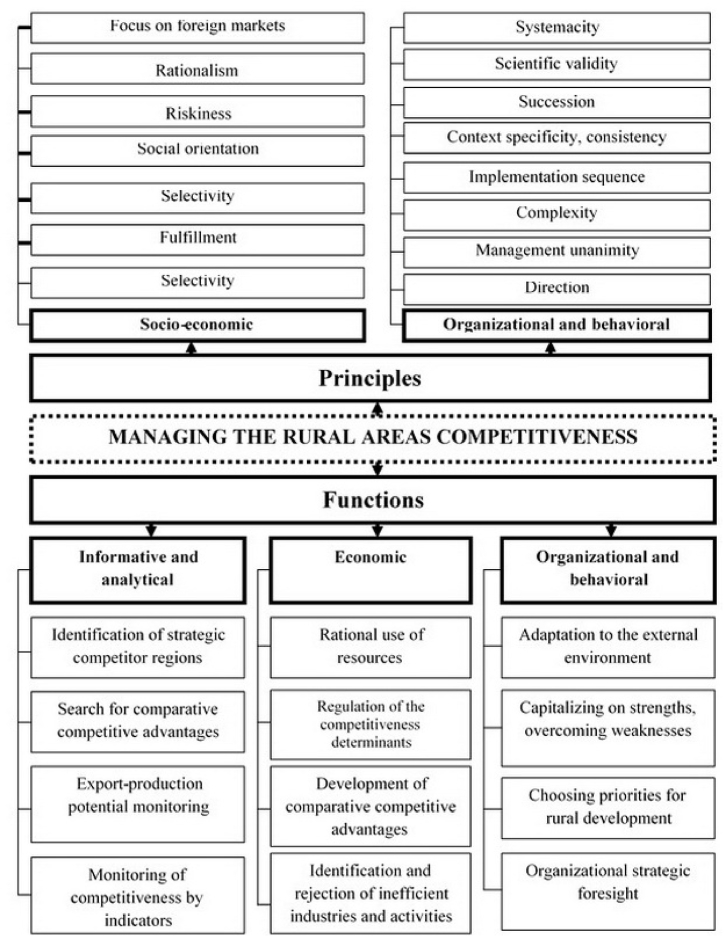
System of principles and functions of management of international competitiveness of rural territories.

*Source:* Authors' development.

According to the research objectives, the second stage, i.e. causal modelling, is of the greatest value. In this stage, researchers commence by interpreting the definition of “international and regional competitiveness of rural areas” through the method of abstraction. This phase is aimed at gaining a deeper understanding of the essence of this category and providing a clear definition of the research subject. Furthermore, within this stage, the principles and functions of managing the regional competitiveness of rural areas are systematized. Additionally, it is crucial to emphasize that, during this phase, a conceptual framework for managing competitiveness is established, integrating a systematic and comprehensive approach that facilitates the examination of connections among different aspects of competitiveness (Fig. 2).

This system is to be the foundation of a conceptual model for managing the international competitiveness of rural areas of the region. The model formation involves a systematic and integrated approach. A systematic approach considers international and regional competitiveness as a set of interrelated subsystems (incoming determinant; process determinant forming competitive advantages; outgoing indicator determinant). An integrated approach reflects all elements of the specified system and its subsystems and assesses the nature and intensity of the interaction. The proposed approach to the management of competitiveness of rural territories involves its assessment using a system of indicators, assessment of their changes according to trend forecasts of changes in the competitiveness determinants, and assessment of changes in indicators by consciously influencing the determinants.

### 3.2 Expert survey

An expert survey was conducted to concretize the conceptual model of managing the international competitiveness of rural territories in the Volga Federal District of the Russian Federation. Fifty expert scientists, heads of agro-industrial enterprises, and representatives of government authorities were interviewed. The survey was conducted online with the following questions.

1. What factors are considered the most important for the competitive advantages of rural areas?
2. What is the role of export production potential in enhancing competitiveness?
3. Which strategic development goals for rural areas do you consider a priority?
4. What internal factors and determinants influence the competitiveness of rural regions?
5. Which external factors and market opportunities are considered for strengthening competitiveness?
6. What tools and strategies are employed for creating competitive advantages?

The survey results allowed the identification of determinants and indicators of the formation of rural territories' competitive advantages depending on the export-production potential and strategic goals of their development.

### 3.3 Competitiveness model

The methodological approach to managing the competitiveness of rural areas using the model is to develop a model that includes a system of incoming factors (determinants) affecting the level of competitiveness level, its dynamics, a three-level process of forming rural regions' competitive advantages and a system of output parameters (indicators) for assessing the level of competitive advantage. This model involves external and internal determinants and macro environments. Indicators are divided into indicators of rural regions' competitive advantages, regional efficiency and export-production potential. The mentioned model allows for assessing the current state of international and regional competitiveness of rural areas and predicting changes in its indicators depending on the regulating of the competitiveness determinants.

The assessment is based on the method of comparative analysis when integral and partial competitiveness indices are determined at each level of comparison. The ordered estimate method allows ranking the results obtained in order of importance. The methodology lies in the fact that each indicator is given the rating of each region according to its position considering the indicator's importance. Summing up the ratings of rural territories of the region by all indicators allows for determining the rural territories that have the maximum competitive advantages over others (it is possible to summarize the ratings adjusted for the importance coefficient of the corresponding indicators). The advantages of the methodology are ease of use and the ability to compare absolute and relative indicators. The integral estimation method provides for the calculation of the integral coefficient as the arithmetic mean of the partial coefficients.

The final step of the second stage is the algorithmization of international competitiveness management considering the above model. The authors developed an algorithm for the formation of competitiveness of rural territories. The algorithm makes it possible to justify rational and effective strategies for the socio-economic development of regions by considering the determinants of competitive advantages, and variability of actions for competitive positioning. The algorithm is based on the system-procedural principle of identifying analytical, strategic and tactical subsystems. The method of algorithmization of the formation of international and regional competitiveness of rural areas is supplemented by the use of the algorithm of methods of integral assessment of competitiveness, the method of comparative assessment, SWOT analysis and others. Determining the strategic directions of regional goods producers' entry into foreign markets requires a strategic analysis of all micro- and macro-environment factors. A basic layout drawing of a SWOT analysis of the international competitiveness of rural regions assuming additions, changes and restrictions has been designed (Table 1).

**Table 1:** Layout drawing of SWOT analysis of the international competitiveness of rural regions^a^.

Potential strengths	Potential opportunities
a adequate financial resources; a the popularity of the territory, regional brands in international markets; a convenient location; a a well-developed competitive strategy; a developed storage/retrieval logistics; a favorable agro-climatic conditions; a developed proprietary technologies; a cost leadership; a extensive experience in foreign economic activity (ahead of the experience curve); a excellent technological skills; a state support for imports and foreign economic activity; a high production and export potential; a others	a ability to enter new international markets; a the ability to adapt products to the requirements of international markets; a reducing trade barriers in attractive foreign markets; a weakening of the positions of competing firms in international markets; a the possibility of rapid development due to a sharp increase in demand in international markets; a the emergence of new technologies; others
**Potential weaknesses**	**Potential threats**
a there is no clear strategic direction for export development; a outdated equipment; a low profitability due to poor agro-climatic conditions; a lack of managerial skills in conducting international business; a low production potential; a lag in research and technologies; a a limited assortment of products; a poor product quality a insufficient image in the international market; a high barriers to entry into international markets; a unsatisfactory organization of external marketing activities; a environmental inadequacy; a others	a entry into the market of foreign competitors with lower costs; a growth in sales of substitute products; a unfavorable changes in the foreign currency rates or the states' trade policies; a expensive legal requirements; a buyers demands in international markets; a changing customer needs and tastes; a adverse demographic changes; a introduction of economic sanctions, customs quotas; a others

a *Source:* Authors' development.

### 3.4 Validation of the results

The final stage is the approbation analysis involving empirical methods and assuming the digitization of methods and models on the example of a specific rural region of one country. The paper presents one of the agricultural regions of Russia, the Republic of Bashkortostan, as an object of approbation. The information and analytical base of this stage was the official data of federal and regional bodies of the Federal State Statistics Service of the Russian Federation (statistical and factual materials), analytical materials of the Federal Customs Service of the Russian Federation, and research results of leading scientists.

## 4 Results

The conducted research allowed authors to develop a new approach to interpreting the term “international, regional competitiveness of rural territories”. By this term, the authors understand the position of rural territories of the region in the domestic and external (international) markets reflected through a comparative assessment of indicators (indicators) and occasioned by production and export potential, competitive advantages, and economic, social and environmental factors (determinants).

**Fig. 3:**
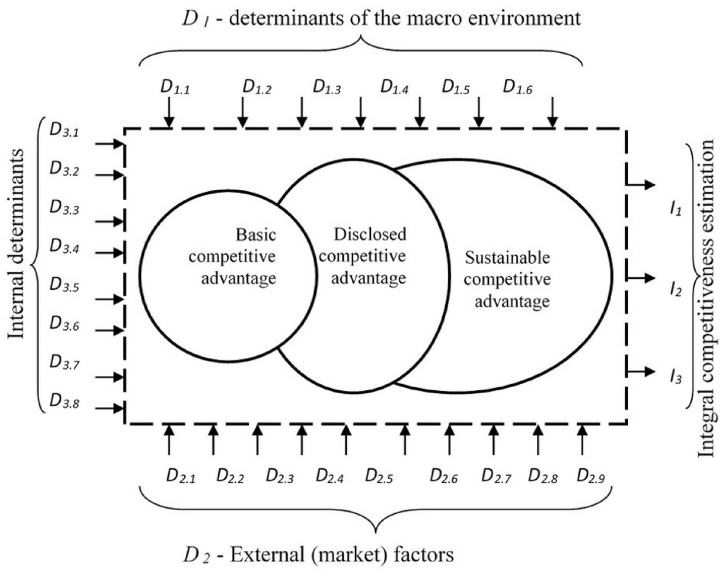
Model of formation and management of regional and international competitiveness of rural areas of the region.

*Source:* Authors' development.

Management of the international competitiveness of rural regions is a systematic and complex process that can be represented as a multi-parametric model (Fig. 3). The basis of the model is a competitive advantage that ensures the competitiveness of rural areas and has three levels-basic, disclosed and sustainable. The diagram reflects the nature of the levels' development. The disclosed advantage increases the size of the basic, and the sustainable one provides a long-term advantage without seriously increasing it. A system of determinants influences the formation of a competitive advantage. Thus, state and municipal authorities control the internal determinants to increase the regional competitiveness of rural areas. The external determinants are regulated; they should be considered, and identified, to use promising market opportunities. The determinants of the macro-environment are uncontrolled but they should be considered when choosing tools for competitiveness formation.

The formation of the international competitiveness of rural areas is influenced by determinants. Determinants are factors that determine and influence this process. Changing determinants lead to a change in the level of rural areas' international competitiveness. The determinants of a region's competitiveness can be manifested in all cognitively significant spheres of the region and beyond its limits. The simplest way to select determinants regarding the region is to use the M. Porter diamond. The determinants are demand parameters in the regional commodity market, parameters and conditions of factors of production, sales and infrastructure, kinship and supporting industries. In the authors' opinion, these determinants do not cover all aspects of the formation of competitiveness of rural territories of the region and do not consider rural specifics. Therefore, the authors propose to expand and systematize the determinants of competitiveness in relation to rural areas. The macro-environment plays a pivotal role in shaping the competitiveness of rural areas. Economic stability, the political environment, and sociocultural changes can exert a substantial influence on agriculture and the agricultural product market. It is essential to comprehend how these macro-factors interact with rural regions and the opportunities they offer.

External market-related factors also play a crucial role in determining the competitiveness of rural areas. The demand and supply of agricultural products, the level of competition, and innovation in production can shape strategies and opportunities for the development of rural regions. Effective responses to external market factors can enhance competitiveness.

Therefore, it is also important to pay attention to internal factors that are under the control of rural areas themselves. Management and strategy, resource availability, infrastructure, the level of agricultural production, and human capital play a significant role in determining competitiveness. Internal factors provide rural regions with tools and opportunities to enhance their competitiveness in the long term (Table 2).

**Table 2:** Determinants of the formation of international and regional competitiveness of rural areas of the region^a^.

Groups of determinants	The main determinants	The impact on the rural ares competitiveness
*D*_*1*_ Macro environment determinants	*D*_*1.1*_ Natural factors	High
	*D*_*1.2*_ Scientific and technical factors	High
	*D*_*1.3*_ Macroeconomic factors	Moderate
	*D*_*1.4*_ Socio-demographic factors	Moderate
	*D*_*1.5*_ Legal factors	Moderate
	*D*_*1.6*_ Cultural, aesthetic factors	Slight
	*D*_*1.7*_ Political factors	Slight
*D*_*2*_ External (market) factors	*D*_*2.1*_ The state of the competitive environment in the top domestic and foreign markets	Moderate
	*D*_*2.2*_ The region's position in national and international markets	High
	*D*_*2.3*_ Current and potential competitor regions	High
	*D*_*2.4*_ Threats of new players (competitors) entering the market	Moderate
	*D*_*2.5*_ Substitute products	Slight
	*D*_*2.6*_ The level of suppliers' power in the market	Moderate
	*D*_*2.7*_ Actual and prospective marketing outlets and channels	Moderate
	*D*_*2.8*_ Market infrastructure and its accessibility	Moderate
	*D*_*2.9*_ The level of buyers' power in the market	Slight
	*D*_*2.10*_ Market environment	High
*D*_*3*_ Internal factors	*D*_*3.1*_ Rural areas production resources	High
	*D*_*3.2*_ The region export potential	Moderate
	*D*_*3.3*_ Location and logistics infrastructure	Moderate
	*D*_*3.4*_ The level of development of the territory marketing system	High
	*D*_*3.5*_ The level of development of agricultural production	Moderate
	*D*_*3.6*_ Image, the popularity of the region and its products	Moderate
	*D*_*3.7*_ The region specialization and its level	Slight
	*D*_*3.8*_ The level of agri-tourism development	Moderate

a *Source:* Authors' development.

The formation of competitiveness is influenced by the determinants of the macro environment, market external and internal factors.

This system of determinants reflects the most significant factors in the formation of the rural areas' international competitiveness in the region.

Managing the international and regional competitiveness of rural areas is impossible without evaluating the effectiveness. The authors have developed a system of indicators - observable and measurable characteristics that allow the conclusion on the level and trends of changes in the comparative competitive advantages of the region (Table 3). The system of competitive advantage indicators includes characteristics and their changes according to three groups of indicators: external (market), internal, and indirect. Table 3 reflects the nature of the relationship between the quantitative values of these indicators and the level of competitive advantage. Direct impact means the increase in the level of international and regional competitiveness of rural areas of the region with the increase in the indicator's value.

**Table 3:** Main indicators of international and regional competitiveness of rural areas of the region^a^.

Groups of indicators	Main indicators	Impact on the competitiveness estimation
*I*_*1*_ Regional performance indicators	*I*_*1.1*_ Gross regional product produced in the agro-industrial complex, per 1 person employed in the economy of the regions	Direct
	*I*_*1.2*_ The level of agricultural production	Direct
	*I*_*1.3*_ Indicators of the value of fixed assets in agriculture, per 1 employed person	Direct
	*I*_*1.4*_ Percentage of the customer churn	Reverse
	*I*_*1.5*_ The share of science-intensive and exported products	Direct
	*I*_*1.6*_ Average per capita investment in fixed assets	Reverse direct
	*I*_*1.7*_ Indicators of dynamics and the ratio of monetary income per capita and 1 ruble GRP per capita in rural areas	Direct
	*I*_*1.8*_ Gross regional product per 1 ruble of the cost of the region main fund (and/or in the agro-industrial complex)	Direct
	*I*_*1.9*_ Indicators of dynamics and the ratio of monetary income per capita and 1 ruble of GRP per capita	Direct
*I*_*2*_ Indicators of competitive advantages of rural regions	*I*_*2.1*_ Indicators of crop and livestock production profitability	Direct
	*I*_*2.2*_ Balanced financial result of crop and livestock production	Direct
	*I*_*2.3*_ Gross grain harvest	Direct
	*I*_*2.4*_ Production of livestock and poultry for slaughter	Direct
	*I*_*2.5*_ Milk production in farms of all categories	Direct
	*I*_*2.6*_ Production of other export-oriented agricultural products and food (according to types)	Direct
	*I*_*2.7*_ Rural areas competitiveness assessment using a static and dynamic SWOT analysis model	Direct
*I*_*3*_ Indicators of export-production potential	*I*_*3.1*_ Economically active rural population of the region	Reverse
	*I*_*3.2*_ The cost of fixed assets for agricultural purposes	Direct
	*I*_*3.3*_ The average number of employees of agricultural enterprises	Direct
	*I*_*3.4*_ Agricultural land area	Direct
	*I*_*3.5*_ Assessment of the dynamics of the balanced financial result of the region	Direct
	*I*_*3.6*_ Investments in rural development	Direct
	*I*_*3.7*_ The share of agriculture the gross regional product	Direct
	*I*_*3.8*_ Exports of agricultural products and food in value terms	Direct

a *Source:* Authors' development.

**Fig. 4:**
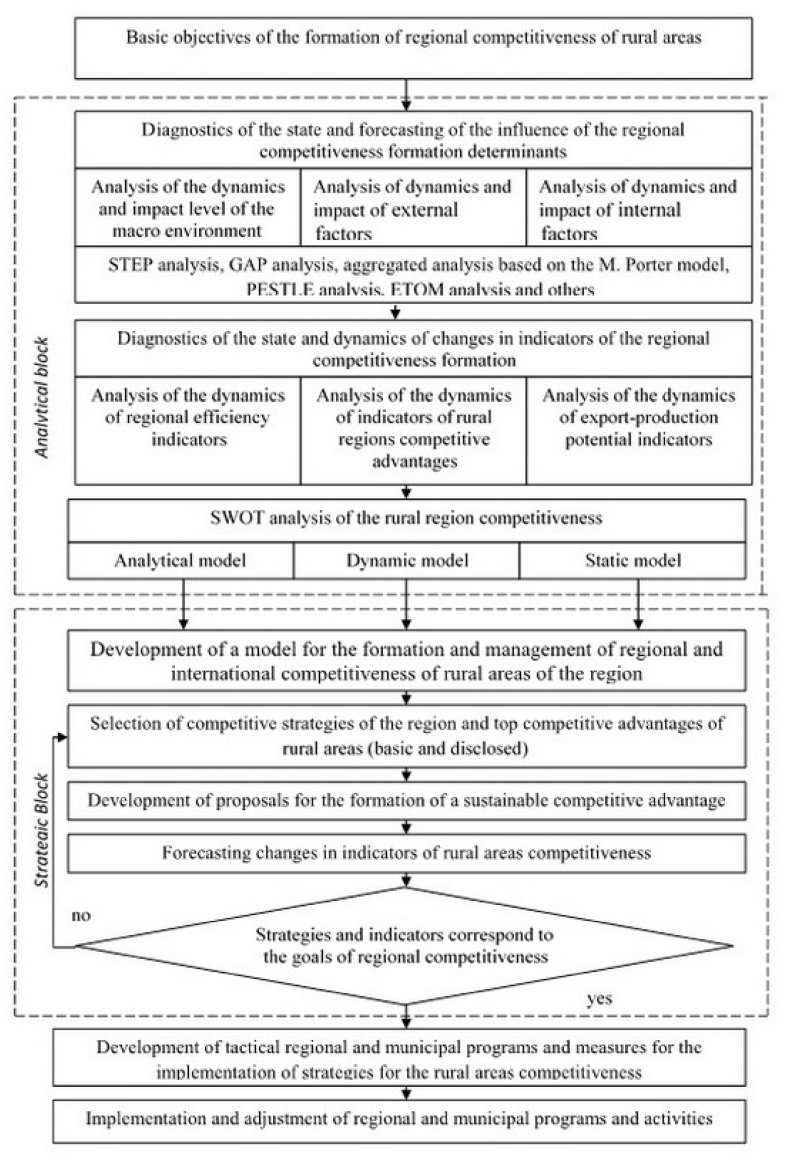
Algorithm of formation of international regional competitiveness of rural territories.

*Source:* Authors' development.

The indicators allow the overall estimation of the enterprise's competitive advantage. The dependence between determinants and indicators (e.g. correlation dependence) of the individual enterprises' activities makes it possible to regulate and manage the formation of the rural areas' competitiveness. To implement this model, the authors have developed an algorithm for the formation of regional competitiveness in rural areas (Fig. 4).

The analytical subsystem supplies the process of competitiveness formation with information. It is a system of data collection and processing. The data collected allows studying the rural areas' competitive position in statics and dynamics, to measure the influence of its determining factors and to identify the region's competitive capabilities. The SWOT analysis is the basis for the strategic subsystem of the algorithm. The subsystem involves the choice of a basic competitive strategy for rural development and top competitive advantages. The most important aspect of the strategic block is the choice of strategies for the formation of the international competitiveness of the region, and the definition and forecasting of indicators of its competitive position.

Steps to forming a sustainable international competitive advantage of the rural region are taken considering the selected international and regional competitive strategies. In the first stage, the formation of this advantage is carried out based on production factors - natural resources, and skilled labor (one determinant). The second stage involves aggressive investment in education, technology, and licenses (three determinants). In the third stage, advantage formation is carried out by creating new types of products, production processes, organizational solutions and other innovations. In the fourth stage - at the expense of already created wealth relying on all the determinants that are not fully used. To influence the internal and external determinants, it is advisable to develop appropriate regional and municipal programs for the development of rural competitiveness.

The mentioned methodological approaches are applied when analyzing the international competitiveness of the regions of the Volga Federal District of the Russian Federation and comparing them with other leading rural regions. The calculation of the integrated indicator of competitiveness was carried out using 24 indicators of competitiveness of rural territories, given in Table 3. The results are presented in Table 4.

**Table 4:** Integrated indicator of the international regional competitiveness of rural territories of Russia.

Regions	Integrated coefficient of international competitiveness		
	2018	2019	2020
The Moscow Region	18.00	16.01	18.00
The Leningrad Region	14.10	19.78	14.48
The Krasnodar Territory - (1st place for the production of agricultural products in the Russian Federation)	74.56	103.90	77.98
The Belgorod region – (3rd place for the production of agricultural products in the Russian Federation)	52.91	64.17	56.64
The Rostov region – (2nd place for the production of agricultural products in the Russian Federation)	45.47	18.74	76.88
The Volga Federal District			
The Republic of Bashkortostan	38.47	24.74	29.23
The Republic of Mari El	7.34	0.21	10.89
The Republic of Mordovia	21.94	22.87	22.00
The Republic of Tatarstan	56.62	35.40	32.76
The Udmurt Republic	23.84	17.11	14.96
The Chuvash Republic	16.95	10.79	5.70
The Perm Region	37.68	11.07	11.85
The Kirov region	18.58	16.40	14.64
The Nizhny Novgorod Region	35.04	16.68	16.32
The Orenburg Region	32.22	18.80	22.45
The Penza Region	19.22	23.06	29.12
The Samara Region	38.93	17.92	22.26
The Saratov Region	35.48	27.36	29.95
The Ulianovsk Region	18.69	10.69	12.20

^a^*Source:* Authors' calculations based on data from the Federal State Statistics Service of the Russian Federation.

Calculations show that the leading regions (the Krasnodar Territory, the Rostov Region, and the Belgorod Region) have the highest values of integral. As for the competitive advantages of the regions, the best indicators belong to agricultural production (the advantages exceed the values of the comparison base by 2.2–3.81 times in different periods), the value of fixed assets for agricultural purposes (186.5 million rubles - 346.4 million rubles in 2018–2020 with the highest value of 520.4–565.9 million rubles for the Moscow Region), the number of people employed in agriculture (according to this indicator, the leaders of regional competitiveness are the Republic of Bashkortostan, the Republic of Tatarstan, the Orenburg Region), the profitability of crop production (maximum values up to 32.3%-55.6%–55.6 % were achieved in 2020 by agricultural enterprises of the Krasnodar Territory, Belgorod and Rostov Regions), gross grain harvest, livestock and poultry production for slaughter, milk, agricultural land area. The agriculture of the Moscow Region has a strong position in the market too. Thus, the value of the integral coefficient of competitiveness in the region is 16.0–18.0 in 2018–2020.

**Fig. 5:**
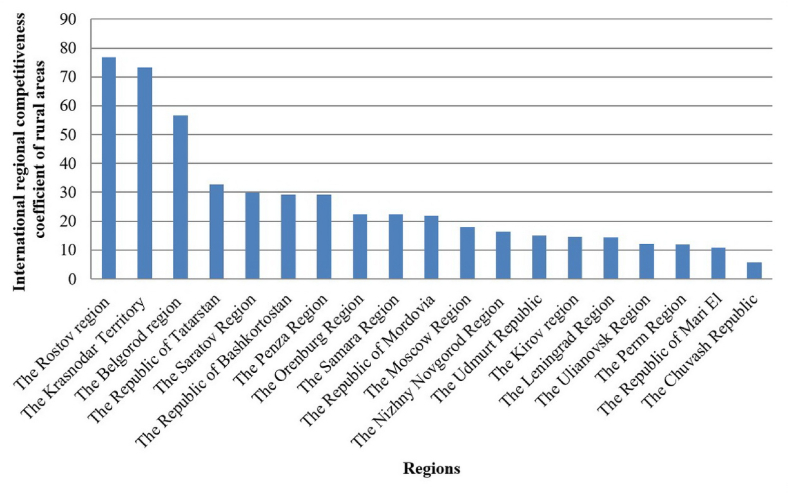
International regional competitiveness of rural areas rating of regions of the Russian Federation (2020).

*Source:* Authors' development.

Among the regions of the Volga Federal District, the Republic of Tatarstan ranks first in the coefficient of international regional competitiveness of 32.76–56.62 in 2018–2020. The same indicator in the Republic of Bashkortostan is 24.74–38.47 and, in the Saratov Region, - 27.36–35.48. It must be admitted that the competitiveness coefficient was decreasing in all regions of the Volga Federal District until 2020. Agricultural enterprises of the Perm Region, Nizhny Novgorod, Samara and Orenburg regions have weak positions except the Penza Region, whose indicator is steadily increasing. Fig. 5 shows the international regional competitive rating.

The Republic of Bashkortostan ranks 6th in the international competitiveness rating of Russia. The analysis of quantitative indicators of the international competitiveness of the Republic of Bashkortostan showed that in 2020 the export of products from the region amounted to 3152.5 million US dollars, which is 26.0 % lower than in 2019, 29.4 % lower than in 2018, and 68.1 % lower than in 2010 (Table 5).

**Table 5:** Export of products of the Republic of Bashkortostan^a^.

Indicators	2010	2018	2019	2020
Exports of goods and services, millions of dollars USA	9890.8	4464.7	4256.8	3152.5
Including export of food products and agricultural raw materials	76.4	76.2	112.1	177.7
Imports of goods and services, millions of dollars USA	749.2	1019.4	764.6	927.8
Including export of food products and agricultural raw materials	23.2	21.0	14.4	25.2

a *Source:* Compiled by the authors according to the data of the Federal State Statistics Service of the Russian Federation.

Though the republic is an agricultural region, its main exporting goods are mineral commodities (39.3 % of the region's exports in 2020) and fuel and energy products (39.2 %). The share of exports of food products and agricultural raw materials in 2020 was only 5.6 %.

**Fig. 6:**
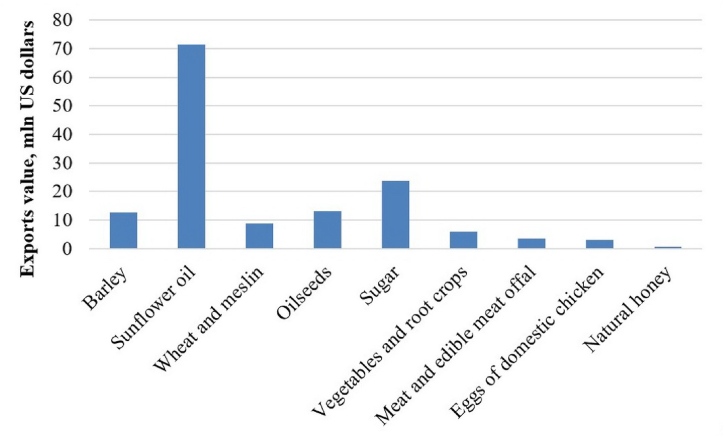
The most competitive goods exported by the Republic of Bashkortostan, 2020.

*Source:* Compiled by the authors according to the data of the Federal State Statistics Service of the Russian Federation.

However, the volume of exports of food products and agricultural raw materials has increased by 2.3 times since 2010. The most competitive goods exported by the Republic of Bashkortostan are (Fig. 6) barley ($12.7 million US dollars in 2020), sunflower oil (71.4 million US dollars), wheat and meslin (8.8 million US dollars), oilseeds (13.0 million US dollars), sugar (23.7 million US dollars), vegetables and root crops 5.9 million US dollars), meat and edible meat offal (3.6 million US dollars), eggs of domestic chickens (3.1 million US dollars), natural honey (0.2 million US dollars).

**Fig. 7:**
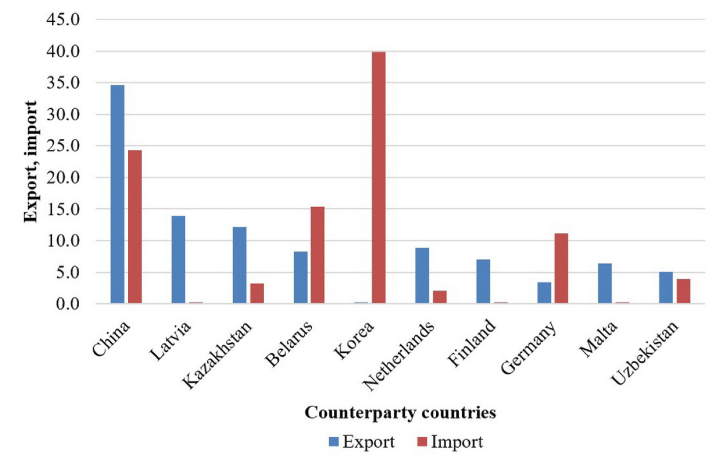
TOP 10 counterparty countries of the Republic of Bashkortostan, million US dollars.

*Source:* Compiled by the authors according to the Federal State Statistics Service of the Russian Federation.

One hundred twenty-four countries were trading partners of the Republic of Bashkortostan in 2020. The largest export trading partners (Fig. 7) are China (25.0 %), Latvia (10.1 %), Kazakhstan (8.8 %), the Netherlands (6.4 %), Belarus (6.0 %), Finland (5.1 %), Malta (4.6 %), Uzbekistan (3.7 %), Turkey (3.0 %), India (2.9 %), Germany (2.5 %), Singapore (2.0 %), the United Kingdom (1.4 %), Lithuania (1.0 %), the United States (1.0 %). The partners in imports are Korea (27.6 %), China (16.8 %), Belarus (10.6 %), Germany (7.7 %), Italy (3.7 %), Japan (3.4 %), the United States (3.3 %), Uzbekistan (2.7 %), India (2.5 %), France (2.3 %), the Netherlands (1.4 %), Belgium (1.4 %).

Besides, the region also exports agricultural products to Canada, Jordan, Saudi Arabia, Mongolia, and Iran. Certain types of the region's products are highly competitive in the markets of these countries. The agricultural products of the region are exported to different countries depending on demand. Food wheat, rye, flax seeds for processing, sunflower meal, oats, feed wheat are exported to Latvia; feed vetch, mustard, buckwheat, flax seeds are exported to Poland; wheat and buckwheat groats, honey are exported to Turkey; goose feather and fluff, flax seeds are exported to Italy; flax seeds, sugar beet pulp are exported to the Netherlands.

At the same time, the relatively low international competitiveness of the region (competitiveness coefficient 29.23 in 2020) is largely due to the barriers to entry to foreign markets, that is, subjective and objective factors preventing regional enterprises from organizing profitable production in other countries or exporting their products to these countries. The most impassable barriers to entry into foreign markets for regional enterprises are sanction restrictions, high transaction costs, lack of sufficient information about foreign markets, legal norms in the quality and ecological properties of products, and availability of marketing channels and logistics.

The strategic SWOT analysis (Table 6) revealed a significant production potential of the republic for the production of agricultural products in demand abroad. The republic is well located geographically and can produce environmentally friendly products. The state support in the region is strengthening including transportation costs.

**Table 6:** SWOT analysis of the international competitiveness of the Republic of Bashkortostan.

Strengths (S)	Weaknesses (W)
Significant energy resources, labor resources at a relatively low cost of labor.	Lack of a well-established export-oriented logistics system
State support for the republic's exports	Strong competitive pressure in the global market
Unique geographical location on the border of Europe and Asia; the intersection of the most important water ways, rail facilities, and highways	Insufficient popularity of the region in the world market; lack of recognizable regional brands
High production potential and activity of the large-commodity sector of the agro-industrial complex	Lack of effective training programs for conducting foreign economic activity
The balance between the extractive, processing and agricultural sectors	Undeveloped financial and organizational infrastructures of foreign trade cooperation
Advanced positions in the production of agricultural products in Russia	Lack of strategic planning and management of the region's international competitiveness
Growth of exports of food and agricultural raw materials	Limited range of exported products, especially ultra-processed products
Products ecological and natural properties	Absence of international quality certificates and difficulties in their obtaining
Moderately favorable natural and climatic conditions	The presence of high price, market and administrative barriers to the market entry
**Opportunities (O)**	**Threats (T)**
Improvement of agricultural production technologies, including digitalization	Tightening of control over quality and ecological properties of products in international markets
Gaining market share from competitors due to product quality, expanded assortment, product promotion	Increased competition for investors and foreign markets between Russian regions
The processing enterprises of the region have unused production capacities	Strengthening of economic sanctions, including restrictions on the import of products to some promising markets
Deeper processing of raw materials, chilled products and increasing the share of such goods in exports.	Unfavorable market conditions due to the pandemic
Active branding and promotion of republican brands to the market	

^a^*Source:* authors' development.

However, some weaknesses significantly hinder export development abroad. They are an undeveloped transport and logistics system, strong competition in world markets, including competition in price proposals because of more favorable natural and climatic conditions and low production costs, difficulties in obtaining international certificates and others. Increasing regional competitiveness involves some measures. They include the complete digitizing of the conceptual model using correlation and regression analysis, determining the desired values of competitiveness indicators by 2030 based on expert strategic sessions using foresight, identifying top measures to improve the international competitiveness of the rural region by optimizing a digitized model with specified indicators and varying determinants within the existing restrictions. Furthermore, these measures may encompass.

• Modernizing transportation and logistical infrastructure.
• Reducing production costs, for example, by enhancing efficiency and employing modern technologies.
• Enhancing accessibility to international certifications and quality standards.
• Developing marketing strategies for promoting products in global markets.
• Exploring new market segments or new products for production diversification.

## 5 Discussion

In the era of globalization, rural regions must aspire to attain regional competitiveness not only within their respective countries but also in international markets. This aspiration entails the adoption and implementation of state, regional, and municipal development programs for foreign economic activities in the agricultural sector, comprehensive support for exports, the cultivation of essential competencies, the establishment of export and logistical infrastructure, and a judicious influence on the determinants of international competitiveness in rural regions' economies. Compared to foreign methods of the formation of rural areas, the international competitiveness of the study was based on.

- general conceptual provisions and methodological approaches that specialists use to assess the international competitiveness of regions and design strategies for the socio-economic development of rural areas having similar problems. The approaches involve consistency, complexity, the use of aggregated indices (indicators) considering the influence of such determinants as infrastructure development, production and export potential, investment activity, development and intensity of foreign trade, socio-economic development, institutional factors, parameters of innovation activity [27,[40], [41], [42]];
- using systematic and integrated approaches, when compared with foreign methods, involving the study of international competitiveness as a set of interrelated subsystems and a comprehensive digital reflection of the elements of this system with an assessment of the interaction nature and intensity [6,37,39].

Summarizing the research of the world's leading scientists on the assessment of the international competitiveness of rural areas, key conclusions on methodological tools and scientific and practical approaches can be formed.

- various scientific and methodological approaches and methods can be used to assess the competitiveness of rural areas (Shift-share methodology; the analysis of comparative advantages, ratings, trade indices, the Porter model and others) [14,15]. Each approach has its advantages and disadvantages, its indications and contraindications to use. The choice of the most appropriate methodology in each particular case depends on the strategic goals for the socio-economic development of rural areas of the region;
- regardless of the applied scientific and methodological approaches and methods, the evaluation results should be objective, specific, clear and understandable [23]. It is not allowed to choose a methodology to justify the desired results or managerial decisions that have already been adopted or lobbied by the state;
- the goals and objectives for the development of rural areas should involve increasing regional competitiveness and forming the international competitiveness of the region. One of the main purposes is to assess the production and export potentials of the regional rural areas and to identify the “growth points” of the agro-industrial complex, agribusiness and socio-economic standard of living in rural areas [19,21].

The method planned for use is based on existing and popular parametric models. The key difference between the proposed author's methodology and existing approaches is the study of competitiveness at three levels, involving the use of general and special techniques. One more difference in the methodology is its ease of use and the possibility of using indicators available for observation and measurement (unlike the index of identified comparative advantages) [[43], [44], [45]]. The indicators are used to describe the level and trends of changes in competitive advantages in reference to regional efficiency, competitive advantages of regions, and export-production potential. Another hallmark is the consideration of the positive or negative effect of the factor and the specifics of export-oriented rural areas.

The proposed model of the regional international competitiveness formation can be compared with similar developments of foreign researchers when using models of international competitiveness as an innovative tool for planning the socio-economic development of rural areas [5,6,42] and when studying institutional mechanisms, government programs, and other determinants that identify the processes being formed and trends of sustainable international competitive advantage of rural areas [9,10,12], thirdly. The model can be also used to make a comparative assessment of the competitiveness of individual regions within the country (regional competitiveness) and in foreign markets [3,41].

The theoretical and practical implications of this research encompass the following aspects.

1. **Theoretical Implications:** This study advances theoretical knowledge in the field of rural region competitiveness management. It introduces a novel methodological approach based on the concept of territorial clusters for sustainable development. This approach may serve as a foundation for further research and the additional development of theoretical models in this domain.
2. **Practical Implications:** The tools and methods developed in this research offer valuable practical solutions for governmental bodies, municipalities, and agro-industrial enterprises. They can be employed for managing and enhancing the competitiveness of rural regions, ultimately contributing to economic growth and socio-economic development.

The core message of this research lies in providing innovative tools for managing the competitiveness of rural regions. This message should be distinctly emphasized to underscore the significance of the study and its potential impact on both practice and academia. Hence, this study bears significant theoretical and practical implications, and its core message, which is the development of novel instruments to enhance the competitiveness of rural regions, should be explicitly and prominently presented.

However, the model has some limitations. For instance, the use of weighting coefficients in the calculation increases the conclusions' subjectivity, and subsequently, the research objectivity decreases. Besides, there are risks of an increase in the average deviations of the results due to an inaccurate assessment of trends and forecasts of changes in determinants. Moreover, this model is recommended only for assessing and managing the international competitiveness of regions and countries with a sufficient development level of market relations, an average and high level of production and export potential of the agro-industrial complex.

The limitation of the study lies in the impact of uncontrollable external factors, such as economic changes, political crises, and natural disasters, which can also influence the outcomes.

## 6 Conclusions

The conducted research has shown that effective management of the international competitiveness of rural areas and the achievement of long-term “desired” indicators is possible when using a reasonable scientific and methodological approach based on the construction of a multi-parametric system model, including interrelated subsystems of determinants, indicators and the multilevel procedural subsystem. The key aspects of the effectiveness of the proposed model are reasonable choice of the model indicators (determinants and indicators), the model digitization considering the conditions of a particular region or market, and algorithmization of actions influencing the procedural subsystem.

Comparative analysis has shown that the proposed conceptual model of managing the international competitiveness of rural areas of the region, methodological recommendations for the model indicators and determinants formation, and the algorithm of actions for the formation of international competitiveness of the region have clearly expressed advantages and correspond to the task of increasing the level of competitiveness and socio-economic development of the region within certain limitations.

The approbation of the developed methodological approaches allowed the following conclusions: rural territories with the highest export and production potential (the Krasnodar Region, the Rostov and Belgorod regions) have the highest rating of international competitiveness in Russia; some regions (the Republic of Bashkortostan) have a low rating of international competitiveness due to the weak use of existing potential. The international competitiveness of the Republic of Bashkortostan is heterogeneous in terms of existing problems and requires the use of various public administration tools. The high production and export potential of the region and trends in changes in the main determinants form significant opportunities for the increase in quantitative and qualitative indicators of the republic's international competitiveness (in particular, ensuring the achievement of the volume of exports of agricultural products in the amount of 228.3 million US dollars the end of 2024).

The studies conducted using the developed algorithm of actions allowed identifying the measures that provide the greatest result in the ratio “cost-effectiveness (level of indicators of international competitiveness)" for the analyzed region.

- formation of sub-regional territorial clusters for the production of priority types of food and agricultural raw materials for export;
- promotion of regional brands and names of places of origin of goods, packaging, and appearance of goods, taking into account the characteristics and mentality of potential foreign markets;
- development of educational programs and training of qualified specialists able to design and implement international projects;
- reducing barriers to foreign market entry by creating rational food chains, the formation of public and private institutions (trade missions, marketing enterprises, fairs, etc.), and the creation of independent organizations to determine the quality of agricultural products.

The study holds significant practical significance for managing the international competitiveness of rural regions and can serve as a foundation for the formulation of strategies and policies aimed at enhancing their competitiveness and socio-economic development.

However, the presented methodological approaches to the formation of international and regional competitiveness of rural areas require further research, especially with regard to the quantitative (digital) description of models for specific regions, methods for quantifying the closeness and nature of the relationships between the elements of the model, the development of a system of assumptions for the use of models. Hence, the prospect of further research is aimed at developing more precise and quantitative models for specific rural territories or regions, as well as exploring ways to integrate the developed methodological approaches into the policies and practices of rural area management. This integration is intended to support their competitiveness and socio-economic development.

## Funding

This research did not receive any specific grant from funding agencies in the public, commercial, or not-for-profit sectors.

## Data availability

The datasets generated during and/or analyzed during the current study are not publicly available due to privacy and ethical restrictions but are available from the corresponding author on reasonable request.

## Ethics approval

All methods were performed in accordance with the principles of the Declaration of Helsinki. The study was approved by Local Ethics Committees of Federal State Budgetary Educational Establishment of Higher Education “Bashkir State Agrarian University” (Protocol № 4 of February 12, 2023).

## Informed consent

Informed consent was obtained from all participants.

## Consent to publish

Not applicable.

## Additional information

No additional information is available for this paper.

## CRediT authorship contribution statement

**Vitaliy Kovshov:** Investigation, Conceptualization. **Milyausha Lukyanova:** Methodology, Formal analysis. **Zariya Zalilova:** Project administration, Data curation. **Oksana Frolova:** Resources, Formal analysis. **Zagir Galin:** Writing – review & editing, Writing – original draft.

## Declaration of competing interest

The authors declare that they have no known competing financial interests or personal relationships that could have appeared to influence the work reported in this paper.
